# *In Vitro* and *In Vivo* Investigation of the Angiogenic Effects of Liraglutide during Islet Transplantation

**DOI:** 10.1371/journal.pone.0147068

**Published:** 2016-03-14

**Authors:** Allan Langlois, Carole Mura, William Bietiger, Elodie Seyfritz, Camille Dollinger, Claude Peronet, Elisa Maillard, Michel Pinget, Nathalie Jeandidier, Séverine Sigrist

**Affiliations:** 1 UMR DIATHEC, EA 7294, Centre Européen d’Etude du Diabète, Université de Strasbourg, Fédération de Médecine Translationnelle de Strasbourg (FMTS), Bld René Leriche, 67200 Strasbourg, France; 2 Service d’Endocrinologie, Diabète, Maladies Métaboliques, Pôle NUDE, Hôpitaux Universitaires de Strasbourg, 67000 Strasbourg, France; Children's Hospital Boston/Harvard Medical School, UNITED STATES

## Abstract

**Introduction:**

This study investigated the angiogenic properties of liraglutide *in vitro* and *in vivo* and the mechanisms involved, with a focus on Hypoxia Inducible Factor-1α (HIF-1α) and mammalian target of rapamycin (mTOR).

**Materials and Methods:**

Rat pancreatic islets were incubated *in vitro* with 10 μmol/L of liraglutide (Lira) for 12, 24 and 48 h. Islet viability was studied by fluorescein diacetate/propidium iodide staining and their function was assessed by glucose stimulation. The angiogenic effect of liraglutide was determined *in vitro* by the measure of vascular endothelial growth factor (VEGF) secretion using enzyme-linked immunosorbent assay and by the evaluation of VEGF and platelet-derived growth factor-α (PDGFα) expression with quantitative polymerase chain reaction technic. Then, *in vitro* and *in vivo*, angiogenic property of Lira was evaluated using immunofluorescence staining targeting the cluster of differentiation 31 (CD31). To understand angiogenic mechanisms involved by Lira, HIF-1α and mTOR activation were studied using western blotting. *In vivo*, islets (1000/kg body-weight) were transplanted into diabetic (streptozotocin) Lewis rats. Metabolic control was assessed for 1 month by measuring body-weight gain and fasting blood glucose.

**Results:**

Islet viability and function were respectively preserved and enhanced (p<0.05) with Lira, *versus* control. Lira increased CD31-positive cells, expression of VEGF and PDGFα (p<0.05) after 24 h in culture. Increased VEGF secretion *versus* control was also observed at 48 h (p<0.05). Moreover, Lira activated mTOR (p<0.05) signalling pathway. *In vivo*, Lira improved vascular density (p<0.01), body-weight gain (p<0.01) and reduced fasting blood glucose in transplanted rats (p<0.001).

**Conclusion:**

The beneficial effects of liraglutide on islets appeared to be linked to its angiogenic properties. These findings indicated that glucagon-like peptide-1 analogues could be used to improve transplanted islet revascularisation.

## Introduction

Clinical islet transplantation has emerged as a promising treatment for type 1 diabetes mellitus, providing stabilisation of glucose metabolism, a sustained decrease in severe hypoglycaemic episodes, restoration of symptom awareness and normalisation of HbA1C levels [1], [2]. However, only 40–50% of patients remain insulin independent 5 years after transplantation [3]. Although the mechanisms involved in graft failure remain elusive, it is believed that 70% of the transplanted islet mass is destroyed in the first few days after transplantation [4], [5]. One of the main causes of this rapid islet loss is insufficient revascularisation of the graft.

After isolation, the re-establishment of blood flow to transplanted islets is essential for their survival; this process requires several days to weeks and involves angiogenesis [1]. Delayed functional microvasculature formation starves islet cells of oxygen and nutrients, causing their apoptosis and/or necrosis [6], [7]. Several studies have demonstrated that vascular endothelial growth factor (VEGF) may be a key protein modulating islet angiogenesis after transplantation [8], [9]. VEGF is synthesized in the pancreatic islets, but its expression in isolated islets is significantly reduced [10], [11]. However, rapamycin, a compound used during the immunosuppressive Edmonton protocol [2], inhibits islet VEGF production and VEGF-mediated survival signalling in tumour cell lines [12]; this could have a major adverse effect on islet revascularisation and may contribute to graft failure over the longer term [13].

Previous studies showed improvements in islet function and vascularisation following *ex vivo* transfection with the human VEGF gene [14], [15]. However, numerous risks have been associated with gene transfer, including the short-term nature of gene therapy, the immune response, problems with viral vectors and the risk of carcinogenesis. Thus, a pharmacological approach to increase VEGF expression may be more suitable for clinical application. We previously reported that pre-treatment of pancreatic islets with deferoxamine, an iron chelator already in clinical use, induced VEGF overexpression by activating the hypoxia inducible factor-1α (HIF-1α) pathway; this approach improved metabolic control in diabetic rats [14], [16]. However, long-term maintenance of graft function was not achieved, probably due to the stabilisation of HIF-1α, which triggers apoptosis at high concentrations [17].

The search for new pharmacological targets capable of stimulating revascularisation of islets led to glucagon-like peptide-1 (GLP-1). Indeed, recent studies revealed that GLP-1 enhanced the proliferation and differentiation of endothelial progenitor cells via upregulation of VEGF [18]. Moreover, exendin-4, a stable GLP-1 receptor agonist with a half-life of 60–90 min [19], also significantly increased the number of new vessels and induced blood flow in Matrigel plugs *in vivo* [20]. Thus, GLP-1 analogues could be used to improve islet revascularisation following transplantation.

The present study was performed using liraglutide, a GLP-1 agonist with a longer half-life (13 h, [19]) than exendin-4, and a greater effect on glycaemic control [21]. Liraglutide was demonstrated to show positive effects on transplanted islets, improving islet survival when administered pre- and post-graft in mice, pigs and humans [22], [21], [23], [24]. However, the effects of liraglutide on islet revascularisation after transplantation have not been investigated.

The aim of our study was to identify whether the beneficial effects of liraglutide on graft survival were due to stimulation of islet revascularisation. Specifically, we aimed to determine the angiogenic properties of liraglutide *in vitro* and *in vivo*, and the mechanisms involved, focusing on the angiogenic targets of HIF-1α and mammalian target of rapamycin (mTOR).

## Materials and Methods

### Animals

All experiments were performed in accordance with the principles and guidelines of the French legislation on animal welfare (Décret 2013–118, 02/01/2013). The proposed experiments were evaluated by French ethical committee number CEEA-35 and the protocols were approved by the French government under authorisations AL/59/66/02/13 and AL/06/35/12/12. Wistar and Lewis rats were supplied by Janvier Labs (Le Genes St Isle, France). Rats were housed in standard collective cages, in a temperature-controlled room (22 ± 1°C) with a 12 h light/12 h darkness cycle. They were fed with SAFE-A04 (Villemoisson-sur-Orge, France). Food and water were available *ad libitum*.

### Islet isolation and culture

Pancreatic islets were isolated from 200-g male adult Wistar rats for the *in vitro* study and from 200 g male adult syngeneic Lewis rats for the *in vivo* study, to sustain immune rejection. The islet isolation procedure was performed using the method described by Sutton *et al*. [25]. Islets were cultured in M199 medium (Gibco^®^, Saint Aubin, France) supplemented with 10% heat-inactivated foetal calf serum (FCS; Sigma-Aldrich, St Louis, MO, USA), and 1% of a mixture of penicillin (10 000 IU/mL), streptomycin (10 mg/mL) and amphotericin B (25 μg/mL) (Gibco^®^). Islets were cultured at 37°C in humidified air with 5% CO_2_ with either 20 per well in 48-well culture plates (Greiner Bio-one, Kremsmünster, Austria) for the function and VEGF secretion studies, or 1000 islets per well in 60 × 15 mm culture dishes (Becton Dickinson, Meylan, France) for protein extraction. Islets were incubated with or without 10 μmol/L liraglutide (Lira, VICTOZA^®^, Novo Nordisk, Denmark) diluted in culture medium for 12, 24 or 48 h. Then, to understand molecular pathway involved by Lira in HIF-1α expression, islets were culture with or without Lira and with or without 100 pg/mL of Rapamicyne (Rapa, Rapamune^®^, Weyth Pharmaceutical, Blois, France), an inhibitor of mTOR during 12h.

Total protein extracts were prepared using M-PER^®^ Mammalian Protein Extraction Reagents (Fisher, Ilkirch, France). Nuclear protein extraction was performed using NE-PER^®^ Nuclear and Cytoplasmic Extraction Reagents (Fisher). Protein was quantified using the Bradford assay [26].

### Islet viability

The current standard method, involving fluorescein diacetate/propidium iodide (Sigma) staining, was used to assess islet viability. Ten islets treated with each of the described conditions were randomly selected by two independent investigators. The ratio between green cells and red cells was used to calculate the percentage of viable islets.

### Islet function

Islets functionality test consists in the incubation of islets in a basal solution containing a low concentration of glucose and then, in a stimulated solution containing a high concentration of glucose. After each step, supernatants were recovered and insulin was titrated. Thus, after 12, 24 and 48h of culture, 10 islets treated with each of the described experimental conditions were handpicked, washed and incubated in Krebs Ringer bicarbonate (KRB) solution containing 10% FCS and 2.5 mmol/L glucose (Basal solution, Sigma). The islets were then stimulated with KRB solution containing 10% FCS and 25 mmol/L glucose (Stimulated solution) for 90 min at 37°C in humidified air with 5% CO_2_. The supernatants were collected and rat insulin was measured using an enzyme-linked immunosorbent assay (ELISA) kit (Mercodia, Uppsala, Sweden). The results were expressed as mg insulin/g of total protein.

### Evaluation of mRNA expression by quantitative polymerase chain reaction (qPCR)

Total cellular RNA was extracted using the RNeasy Plus Mini Kit (Qiagen, Courtaboeuf, France) according to the manufacturer’s instructions. The extracted mRNA (1 μg) was converted to cDNA using a RT² First Strand Kit (SABiosciences, Frederick, MD, United States). qPCR was performed using QuantiTect^®^ SYBR^®^ Green PCR (Qiagen) on a My IQ™5 system (Bio-Rad, Marne-la-Coquette, France). Primer sequences and cycle protocols were obtained from Qiagen (Courtaboeuf, France). Following primers were studied: β-actin (Rat Actb: PPR06570C), HIF-1α (Rat Hif1α: PPR45087B), VEGF-A (Rat Vegfa: PPR06748C) and PDGF-α (Rat Pdgfα: PPR06694C). Relative changes in gene expression were analysed using the 2^-ΔΔCT^ method [27]. Differences in the amounts of template cDNA in each reaction were normalised to the cycle threshold (Ct) value for β-actin. The normalised values were divided by the calibration (t = 0 h) to generate relative expression levels.

### VEGF protein secretion

VEGF was quantified in islet supernatants using a rat VEGF ELISA kit (Tebu-bio, Le Perray-en-Yvelines, France). Results were expressed as pg VEGF/μg total protein.

### HIF-1α and mTOR phosphorylation analyses using western blotting

Western blotting was performed as described previously [16] using the appropriate primary and secondary antibodies: anti-rat HIF-1α mouse monoclonal antibody (R&D Systems, Minneapolis, USA; 1/1000); anti-rat mTOR and phosphorylated mTOR (Ser2448) rabbit polyclonal antibodies (Cell Signalling, Ozyme, St Quentin Yvelines, France; 1/500); anti-mouse goat peroxidase (HRP)-conjugated and anti-rabbit goat peroxidase (HRP)-conjugated antibodies (Sigma-Aldrich, St Louis, MO, USA; 1/8000). Proteins were visualized with the Luminata^™^ Forte Western HRP Substrate (Millipore) and detected using enhanced chemilumiscence (Bio-Rad). The integrated optical density of each band was quantified using the ImageJ v.1.34 software and normalised against β-actin (anti-mouse β-actin monoclonal antibody, 1/10000, Santa Cruz Biotechnology Inc., CA, USA; or anti-rabbit β-actin polyclonal antibody, 1/10000, Abcam, Paris, France).

### Histological study

Immunofluorescent analyses were performed on 4-μm frozen sections of free islets (24 hours post treatments) or of livers transplanted with islets (30 days after graft). The angiogenic properties of liraglutide were determined using a mouse anti-rat cluster of differentiation 31 (CD31) antibody (1/200; BD Biosciences^™^, Le Pont de Claix, France) and the insular structure of the islets was examined using a rabbit anti-rat insulin antibody (1/100, Cell Signaling). The appropriate secondary antibodies (Alexa Fluor^®^ 555 donkey anti-rabbit IgG [H+L] [1/1000, Invitrogen, Fisher, Ilkirch, France] and Alexa Fluor^®^ 488 donkey anti-mouse IgG [H+L] [1/1000, Invitrogen]) were used to visualise these signals and the appropriate positive and negative controls were performed. Fluorescence intensity was measured by microscopy and analysed by NIS-Elements Br Software (Nikon Instruments Inc., Champigny-sur-Marne, France). Ten different islets per condition were measured and data were expressed as the mean value of fluorescence intensity reported to analysed surface ± the standard error of the mean (SEM).

### Islet transplantation

Islet grafting was carried out in male Lewis rats (200 g). Diabetes was induced by a single intraperitoneal injection of 75 mg/kg streptozotocin (Sigma) diluted in a citrate buffer (pH = 4.2); this induced hyperglycaemia within 3 days. Animals were considered diabetic after two consecutive blood glucose measurements of ≥3 g/L, using the Glucose RTU^®^ test (Biomérieux SA, Craponne, France). To control diabetic state of rats, the measure of C-peptide was preferred rather than the measure of insulin to evaluate insulinemia using a rat C-peptide ELISA kit (Mercodia, Uppsala, Sweden) and expressed in pmol/L. Three study groups were included, with 6 animals per group. These consisted of diabetic rats that underwent a laparotomy and an intraportal injection of CMRL (SHAM group), diabetic rats with transplanted islets (control group) and diabetic rats with transplanted islets that were pre-incubated with 10 μmol/L liraglutide (Lira group). The islets were cultured for 24 h prior to transplantation. Transplantation was performed by counting 1000 islet equivalents per kg body-weight, washing these and injecting them intraportally in 1 mL CMRL.

Diabetic rats received one subcutaneous injection of 2 IU insulin in sterile physiological serum daily (Lantus SoloStar^®^ with 100 IU/mL insulin glargine, Sanofi, Paris, France) until achievement of a glucose level of ≤2 g/L post-transplantation.

At the end of the study, euthanasia was performed on rat using a single intraperitoneal injection at 50mg/kg of Doléthal^®^ (54.7mg/mL).

### Metabolic follow up

Metabolic control was monitored for 30 days after transplantation. Body-weight gain was expressed as a percentage of the weight measured at the beginning of the study. Blood glycaemia was determined in plasma after an 8 h fast using the glucose RTU^®^ test (Biomérieux SA, Craponne, France) and expressed as μmol/L.

### Statistical analysis

Statistical tests were performed using STATISTICA^®^ version 10, Statsoft. Differences between two groups were evaluated using a t-test, after testing data normality. Differences between three or more groups were evaluated using an analysis of variance (ANOVA), followed by a LSD comparison. Data were reported as mean ± SEM for the indicated number of replicates and a p value of <0.05 was considered statistically significant.

## Results

### Islet viability and function

**Fig 1:**
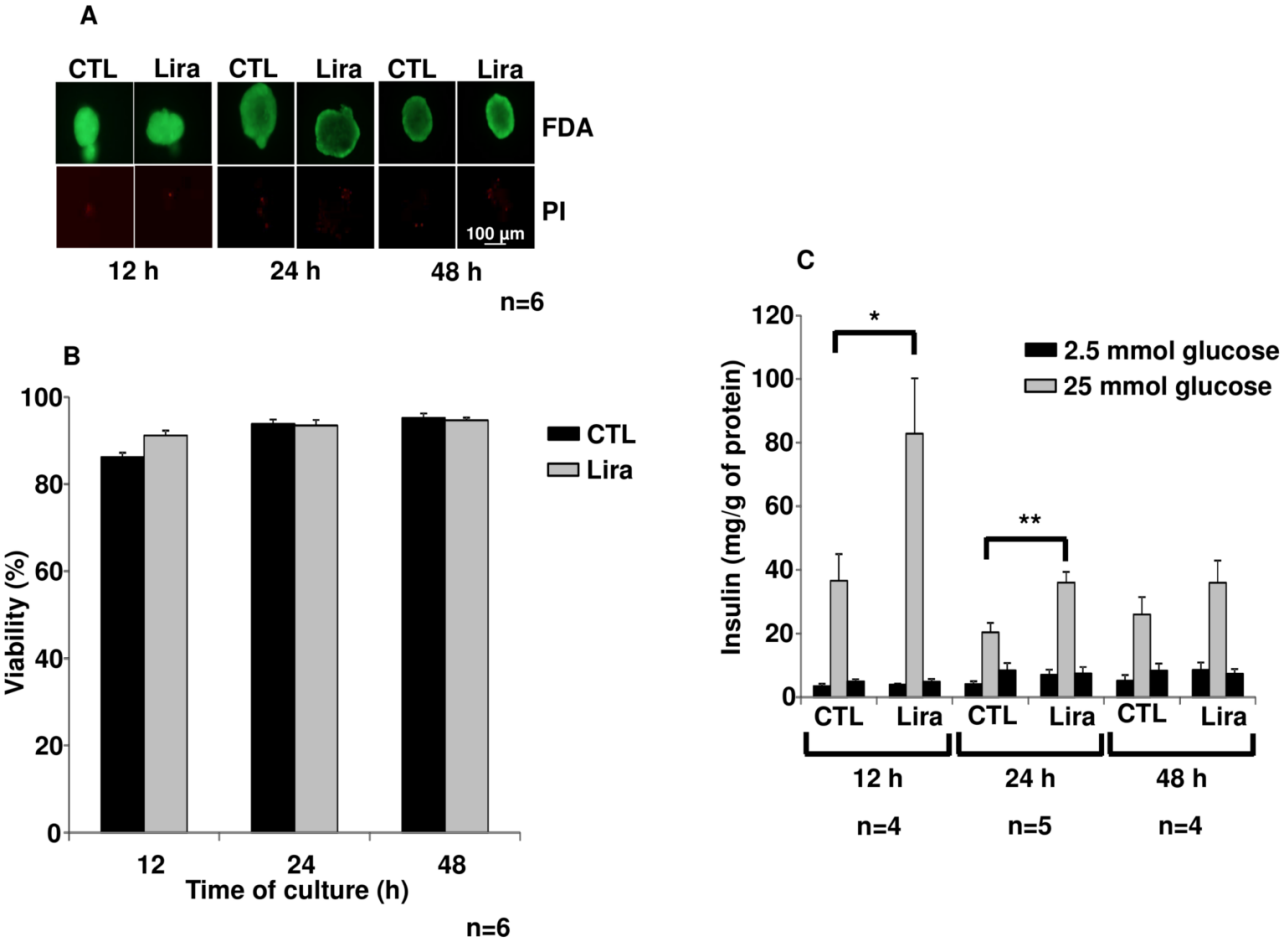
Islet survival and function. (A) Fluorescein diacetate/propidium iodide staining of control islets (CTL) and of islets treated with 10 μmol/L of liraglutide (Lira) at the indicated time-points. (B) Islet viability was also represented in percentage as histograms with controls (black bars) *versus* Lira (grey bars) (C) Glucose stimulation test: Insulin secreted by islet incubated in conditioned medium containing 2.5 mmol/L (black bars) and 25 mmol/L (grey bars) of glucose. Results were expressed as mean ± SEM. *p<0.05, **p<0.01 for the indicated comparisons.

Islet viability was preserved after 12 h, 24 h and 48 h treatments with Lira as compared to control (Fig 1A and 1B).

**Fig 2:**
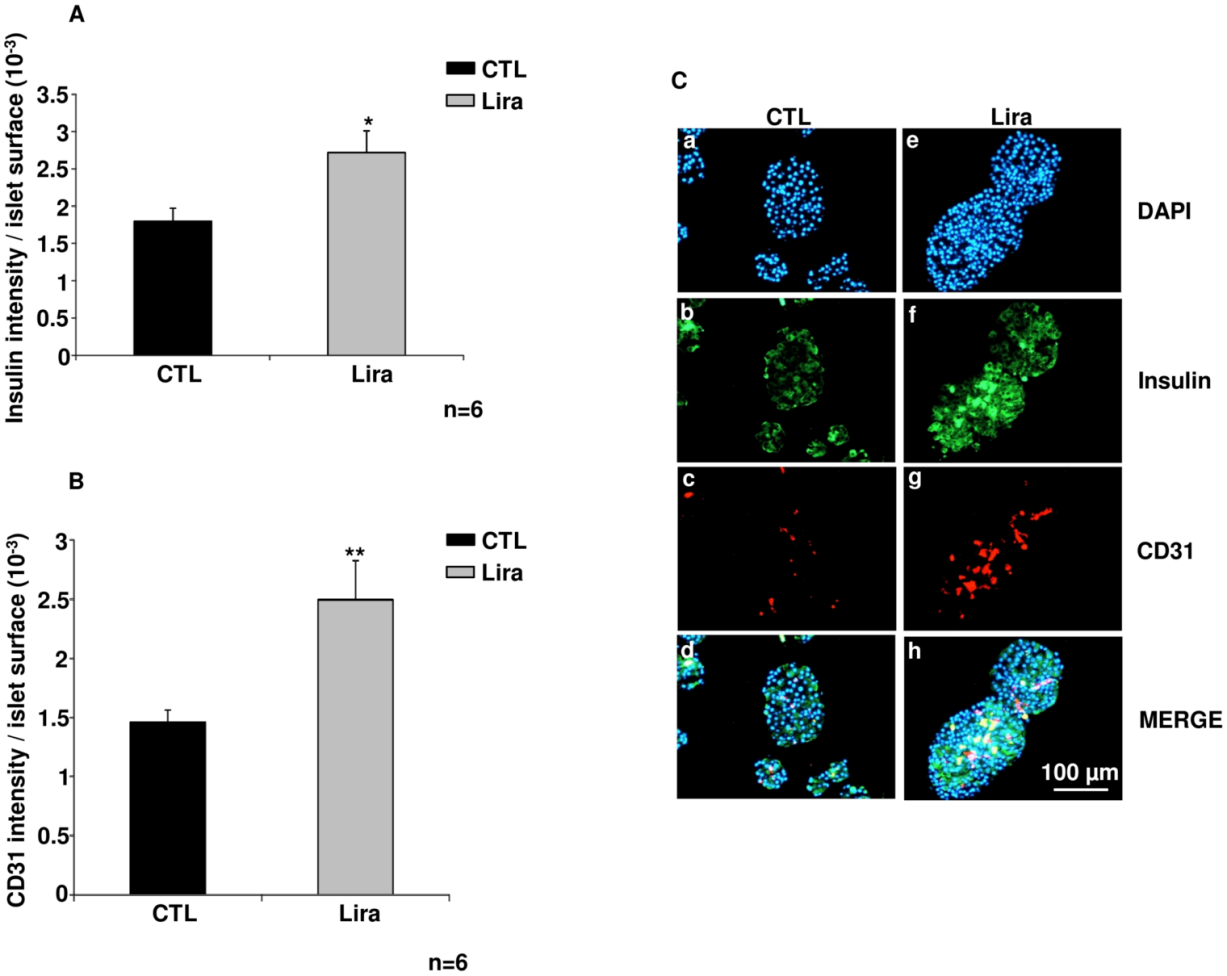
The angiogenic effects of liraglutide *in vitro*. (A) Insulin intensity toward islet surface in control islets (CTL; black bar) as compared to islets treated with 10 μmol/L of liraglutide (Lira; grey bar) (B) CD31 intensity toward islet surface in CTL (black bar) *versus* Lira (grey bar) group (C) Immunostaining of insulin and endothelial cells after 24 h for control islets (CTL; a, b, c, d) *versus* islets cultured with Lira10μM (Lira; e, f, g, h). Nuclear DAPI staining is shown in blue (a, e); insulin staining in green (b, f); vessels are stained red (c, g); and these are merged in d and h. Results were expressed as mean ± SEM. *p<0.05, **p<0.01 for the indicated comparisons.

Islet function was also investigated (Fig 1C). Stimulated insulin secretion was significantly higher after culture with Lira for 12 h (p<0.05, n = 4) and 24 h (p<0.01, n = 6), as compared to untreated islets. After 48 h, the stimulated insulin secretion decreased in islets incubated with Lira and was similar to control levels. Moreover, insulin staining, in green (Fig 2A and 2C) was increased after 24 h Lira treatment (Fig 2Cf), as compared with control (Fig 2A and 2Cb; p<0.05, n = 6). This data is associated to a preservation of insular structure with Lira versus control. Finally, DAPI staining seemed to show more fragmentation in untreated islet as compared to treated islet (Fig 2Ca and 2Ce).

### Angiogenic effects of liraglutide *in vitro*

CD31-positive cells, in red, (Fig 2C) were more prominent after 24 h Lira treatment (Fig 2Cg), as compared with control islets (Fig 2Cc; p<0.01, n = 6). This data was confirmed with the higher CD31 intensity measured in Lira group versus control (p< 0.01, Fig 2B).

**Fig 3:**
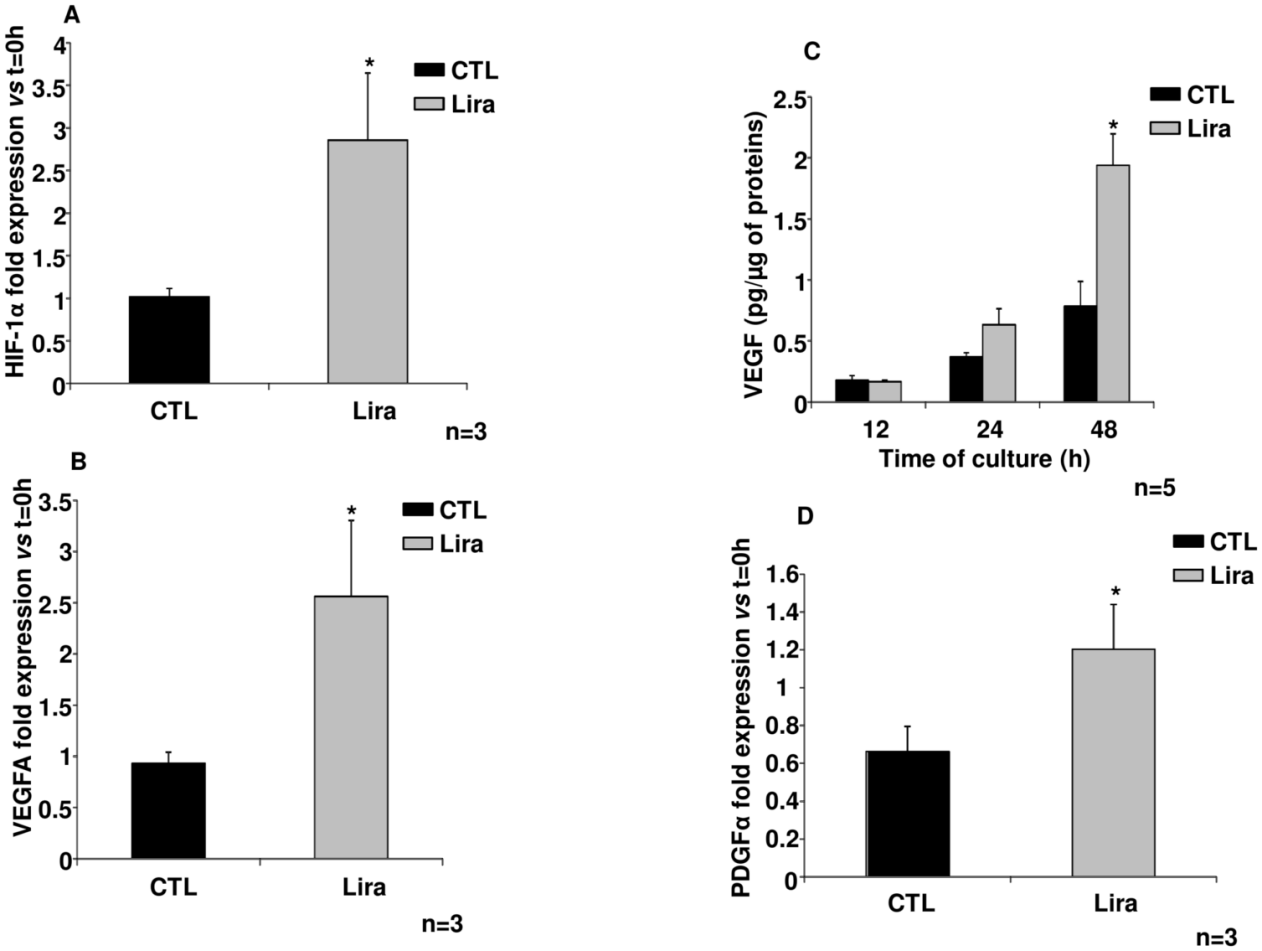
Evaluation of angiogenic markers. (A) Hypoxia-inducible factor-1α (HIF-1α) expression, evaluated by qPCR after 12 h under the indicated conditions and (B) Vascular endothelial growth factor (VEGF) gene expression, determined by quantitative polymerase chain reaction (qPCR) after 12-h exposure to 10μmol/L of liraglutide (Lira) or control (CTL) treatment. (C) VEGF secretion after 12, 24 and 48 h exposure to the indicated treatments using an enzyme-linked immunosorbent assay kit. (D) Platelet-derived growth factor-α (PDGF-α) gene expression, determined by qPCR after 12 h. Black bars, control; grey bars, Lira. Results were expressed as mean ± SEM. Statistics were expressed by * or using # when it is compared respectively to control or to Control + Rapa; *; # p<0.05.

To investigate the angiogenic effects of Lira, the expression levels of genes with known angiogenic properties were evaluated; VEGF, HIF-1α and platelet-derived growth factor (PDGF). Lira treatment of islets increased expression of HIF-1α after 12 h in culture, as compared to control (p<0.05; n = 3; Fig 3A). This result was also confirmed by the significantly elevated nuclear translocation of HIF-1α protein (S1 File) in the presence of Lira for 12 h, *versus* control (Figure A in S1 file, p<0.05, n = 5). HIF-1α translocation returned to the control level after 24 h and 48 h incubations with Lira. In addition, mTOR pathway inhibition using Rapa did not change the level of HIF-1α expression after 12h of culture (Figure B in S1 file). Moreover, a significant overexpression of the VEGF gene was observed after 12 h exposure to Lira, as compared to control, with respective fold increases (compared with t = 0 h) of 2.56±0.74 and 0.93±0.11 (p<0.05, n = 3; Fig 3B). This finding was confirmed by our observation of an elevated VEGF release (Fig 3C) in the presence of Lira for 48 h, *versus* control conditions (p<0.05, n = 6). Finally, PDGF-α expression was only increased after 12 h exposure to Lira (p<0.05, n = 3; Fig 3D).

### Angiogenic mechanisms activated by liraglutide

**Fig 4:**
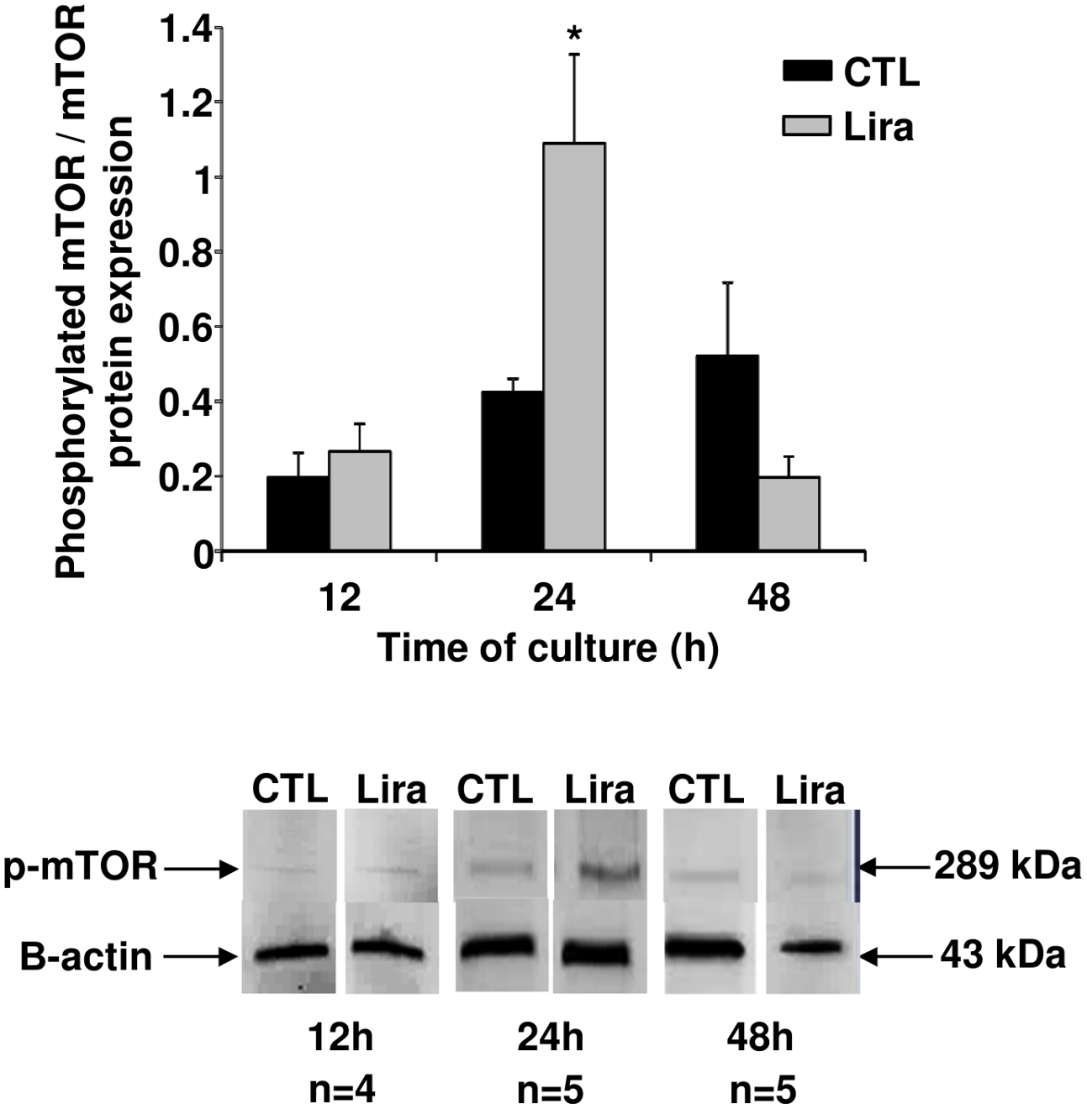
Angiogenic mechanisms induced in rat pancreatic islets by 10 μmol/L of liraglutide. Evaluation of mammalian target of rapamycin (mTOR) activation, determined by the ratio of phosphorylated mTOR/mTOR using western blotting at the indicated time-points. Black bars, control; grey bars, Lira10μM. Results were expressed as mean ± SEM. *p<0.05.

We investigated mTOR pathway activation in cultured islets for 48 h (Fig 4). A significant activation of the mTOR pathway was observed after 24 h exposure to Lira10μM (p<0.05, n = 5). However, this activation had decreased to control levels after 48 h (n = 6).

### *In vivo* investigation of liraglutide efficacy after islet transplantation

**Fig 5:**
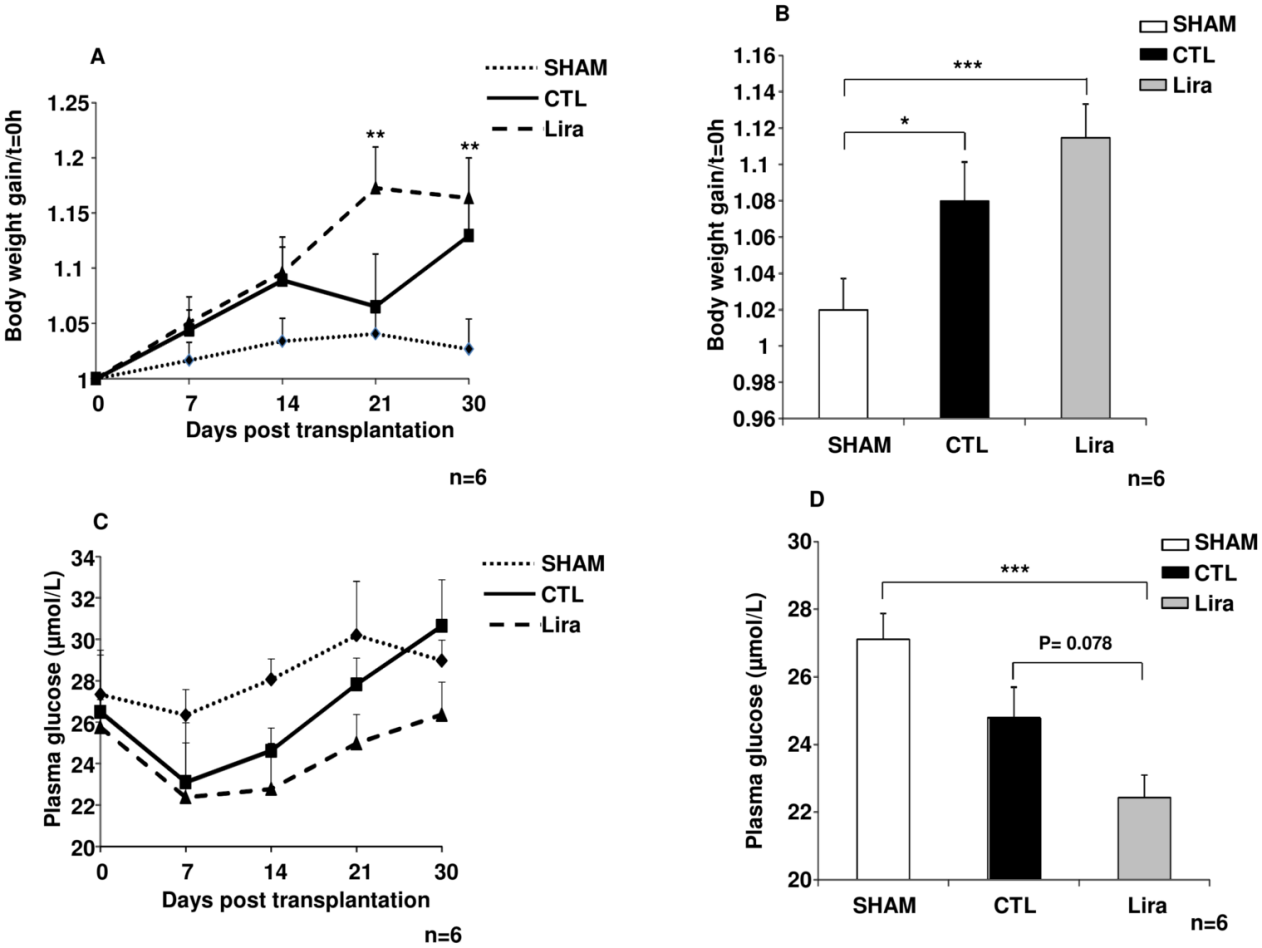
Metabolic control in diabetic rats after islet transplantation. (A) Body-weight gain *versus* t = 0 day in no transplanted diabetic rats called SHAM (filled diamond), in transplanted diabetic animals with untreated islets called control group (filled square) and in transplanted diabetic rats using treated islets with Lira called Lira group (filled triangle). (B) The mean body-weight gains in SHAM (white bar), control (CTL; black bar) and 10μmol/L of liraglutide (Lira; grey bar) groups over the entire experiment. (C) Fasting glycaemia in the SHAM (filled diamond), control (filled square) and Lira (filled triangle) groups at the indicated time-points. (D) Mean fasting glycaemia in SHAM (white bar), control (black bar) and Lira (grey bar) during the experiment. Results were expressed as means ± SEM. ***p<0.001; **p<0.01; *p<0.05.

Body-weight gain and fasting glycaemia were measured in order to assess the metabolic efficiency of islet grafting. Islet transplantation increased body-weight gain in the control and Lira groups, as compared to the SHAM group (n = 6, Fig 5A). This gain was significantly higher in the Lira group than in the SHAM group at 21 days post-transplantation (p<0.01, n = 6) and this difference was maintained at 30 days (p<0.01 *versus* SHAM, n = 6). An analysis of body-weight gain over the course of the experiment (Fig 5B) for each studied group showed a significant increase in body-weight in control rats (p<0.05, n = 6) *versus* the SHAM group and confirmed the higher body-weight gain in the Lira group (p<0.001, n = 6).

Fasting glycaemia (n = 6, Fig 5C) was decreased after islet transplantation, in comparison to SHAM animals, immediately after transplantation. This improvement seemed to be maintained throughout the experimental period in the Lira group. Blood glucose levels had increased in control rats by 21 days post-transplantation, returning to the level observed in the SHAM group. Moreover, statistical analysis of fasting glycaemia in each group during the whole experimental period (Fig 5D) showed that blood glucose levels were significantly lower in the Lira group *versus* the SHAM group (p<0.001, n = 6), while no significant improvement was obtained in the control group. Moreover, fasting glycaemia was reduced more markedly in the Lira group than in the control group (p = 0.078, n = 6).

**Fig 6:**
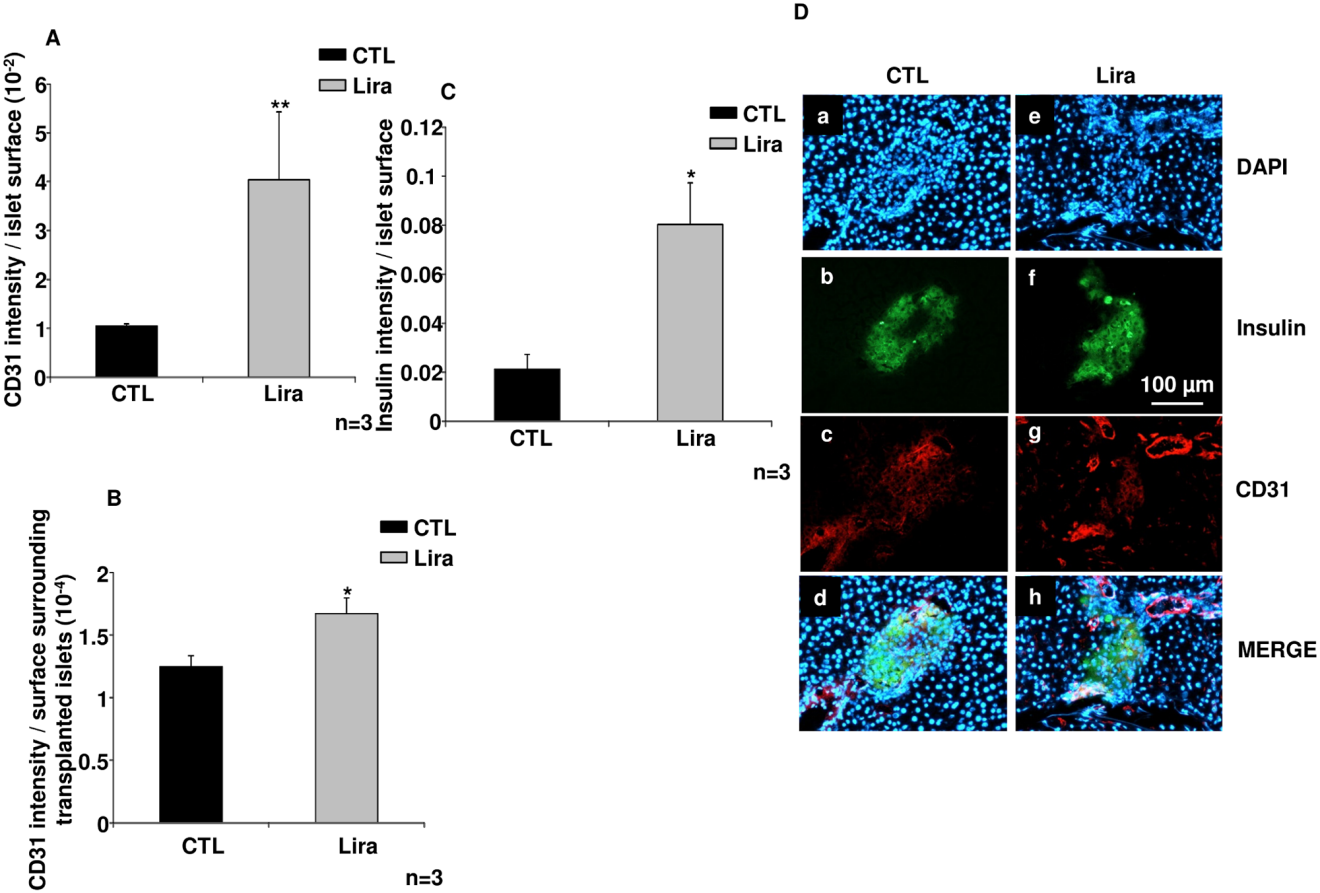
The angiogenic effects of liraglutide *in vivo*. (A) CD31 intensity toward islet surface of rats transplanted with control islets (CTL; black bar) as compared to animals grafted with islets treated with 10 μmol/L of liraglutide (Lira; grey bar) (B) CD31 intensity surrounding transplanted islets toward analysed surface in CTL (black bars) *versus* Lira (grey bars) groups (C) Insulin intensity toward islet surface in CTL islets (black bars) as compared to Lira (grey bar) (C) Immunostaining of insulin and endothelial cells 30 days post implantation for control islets (CTL; a, b, c, d) *versus* Lira (e, f, g, h). Nuclear DAPI staining is shown in blue (a, e); insulin staining in green (b, f); vessels are stained red (c, g); and these are merged in d and h. Results were expressed as mean ± SEM. *p<0.05, **p<0.01 for the indicated comparisons.

Immunohistological analyses of transplanted islets (Fig 6) showed an increased number of endothelial cells inside islets (p< 0.01, n = 3; Fig 6A and 6D) and surrounding transplanted islets (p<0.05, n = 3; Fig 6B and 6D) pretreated with Lira, as compared to the control group. Finally, these increases were associated with improved preservation of the insular structure of the islets (p<0.05 *vs* control, n = 3; Fig 6C, 6Db and 6Df) and a higher nucleus labelling using DAPI in Lira group (Fig 6Da and 6De).

## Discussion

This study identified angiogenic effects of Lira in islets *in vitro* due to activation of the mTOR molecular pathway. This beneficial effect was confirmed by the *in vivo* study, which identified improved islet revascularisation and metabolic control post intraportal transplantation in diabetic rats. Moreover, this work emphasised the great importance of preparing islets appropriately to ensure effective implantation and improved vascularisation, by inducing the expression of angiogenic growth factors such as VEGF.

In addition to the well-characterised anti-apoptotic [28] and antioxidant properties [29] of GLP-1 analogues, we demonstrated that Lira also exerted powerful angiogenic effects on islet grafts. Indeed, *in vitro* and *in vivo* immunostaining studies showed a higher vascular density in the presence of liraglutide, associated with a preservation of insular structure, as compared to control islets. This suggested that pre-treatment with GLP-1 analogues could improve the reestablishment of islet vascular networks after transplantation; this is important because efficient perfusion of the islet core is central to their function and survival [10], [3], [1]. Moreover, our *in vivo* CD31 staining demonstrated that angiogenic pre-transplant signals induced by Liraglutide pre-treatment of islets generated the best vascularisation network at the implantation site. Therefore, these results highlight the important role played by the donor’s angiogenic signals on graft revascularisation and function.

The angiogenic effects observed in the present study was related to the high concentration of liraglutide employed. Indeed, Nishimura *et al* [30] investigated the use of 1 μmol/L liraglutide for islet pre-treatment and subsequently treated the recipients with 100 μg/kg daily subcutaneously after transplantation, as compared to classical intraportal islet graft. No improvement in islet revascularisation was shown. Previously, we have tested *in vitro* on beta cell line Rinm5F, several doses of the molecule. We chose 10 μmol/L and 24h of culture because until this concentration and this time, no decrease of cell viability and higher VEGF secretion *versus* 1 μmol/L were observed (data not shown). In addition, we showed that Lira was not toxic to the islets and improved their function, as described in the literature for lower concentrations [23], [24]. Indeed, our study showed more fragmentation *in vitro* in untreated islet as compared to treated islet. Then, for the in vivo study, we showed a decrease of nucleus labelling without Lira suggesting that the cells are already dead. This anti-apoptotic effect was previously described by Toso C *et al* [24] who obtained *in vitro* a decrease of apoptosis with 1μmol/L of liraglutide in islets *versus* control using TUNEL assay. Moreover, we demonstrated that Lira pre-treatment of islets in culture was sufficient to improve their revascularisation after transplantation, if a supra-physiological concentration was used.

To investigate the mechanisms involved in the angiogenic effects of Lira, we were interested in the following major angiogenic markers: HIF-1α [14], [31] and mTOR activation [32]. Firstly, the angiogenic property of GLP-1 can be explained by its induction of HIF-1α nuclear translocation, increasing VEGF expression and therefore angiogenesis [33], [14], [16]. Lira induced a transient increase in HIF-1α translocation, which seemed to promote islet survival. Indeed, the literature reports that chronic HIF-1α stabilisation in islets induces cell death due to pro-apoptotic signalling [34]. Moreover, Stokes *et al* demonstrated that increasing HIF-1α in a non-toxic manner improved the outcomes for human islets transplanted into SCID mice [35].

We also found that the angiogenic effects of Lira involved the mTOR pathway. Activation of this pathway was linked to the increasing of VEGF secretion *in vitro* and to the higher quantity of endothelial cells observed *in vitro* and *in vivo*. Indeed, the latter is known to play a key role in numerous cellular functions, including endothelial cell proliferation [36]. In the literature, the PI3kinase/AKT/mTOR pathway is reported to contribute to increases HIF-1α levels [37]. Surprisingly, our study observed an increased level of HIF-1α in islets cultured with liraglutide prior to the stimulation of mTOR activation. Thus, this GLP-1 analogue may activate a mechanism that is independent of the PI3kinase/AKT/mTOR pathway, and is responsible for the transient elevation of HIF-1α and to the resultant effects on angiogenesis. We confirmed this hypothesis by inhibiting mTOR using rapamycin, in the presence and absence of Lira. A significant reduction of mTOR activation was observed, with a preservation of the liraglutide-induced increase in HIF-1α translocation at 12 h, in comparison to islets treated with liraglutide alone. Thus, the angiogenic effects of GLP-1 analogues may be due to mTOR pathway activation. Moreover, HIF-1α and mTOR pathway could be interesting to target during islets culture process to improve islet vascularization post implantation and thus, graft survival.

In conclusion, the present study has confirmed the great potential of GLP-1 analogues to improve islet survival after grafting. We demonstrated that the angiogenic properties of Lira could explain its beneficial effects on islet graft survival. We have also confirmed the importance of the rapid restoration of a functional vascular network, ensuring an efficient perfusion of islets. These data reinforce the idea that GLP-1 analogues improve islet revascularisation after grafting. Thus, it would interesting to evaluate the angiogenic effects of Lira in humans, both as a pre-treatment for islets and for administration to recipients during transplantation. Then, the addition of Lira in islet culture medium could represent a new strategy to improve their revascularization after graft. Finally, this pharmacological approach could be associated to immunological strategies [38] [39] for the establishment of a safe and effective protocol to optimize islet transplantation.

## Supporting Information

S1 File Evaluation of angiogenic markers. HIF-1α nuclear protein levels, determined by western blotting after 12, 24 and 48 h in culture. Black bars, control; grey bars, Lira (Figure A) HIF-1α nuclear protein levels, determined by western blotting after 12h in culture. Black bars, control; grey bars, Lira; striped bars, control + Rapa; grey bars with dotes, Lira + Rapa (Figure B).
(TIF)
