# Supplementary figures and images for: In Vitro and In Vivo Investigation of the Angiogenic Effects of Liraglutide during Islet Transplantation
Source: PLoS One. 2016 Mar 14;11(3):e0147068. doi: 10.1371/journal.pone.0147068 (PMC4790919; doi:10.1371/journal.pone.0147068)

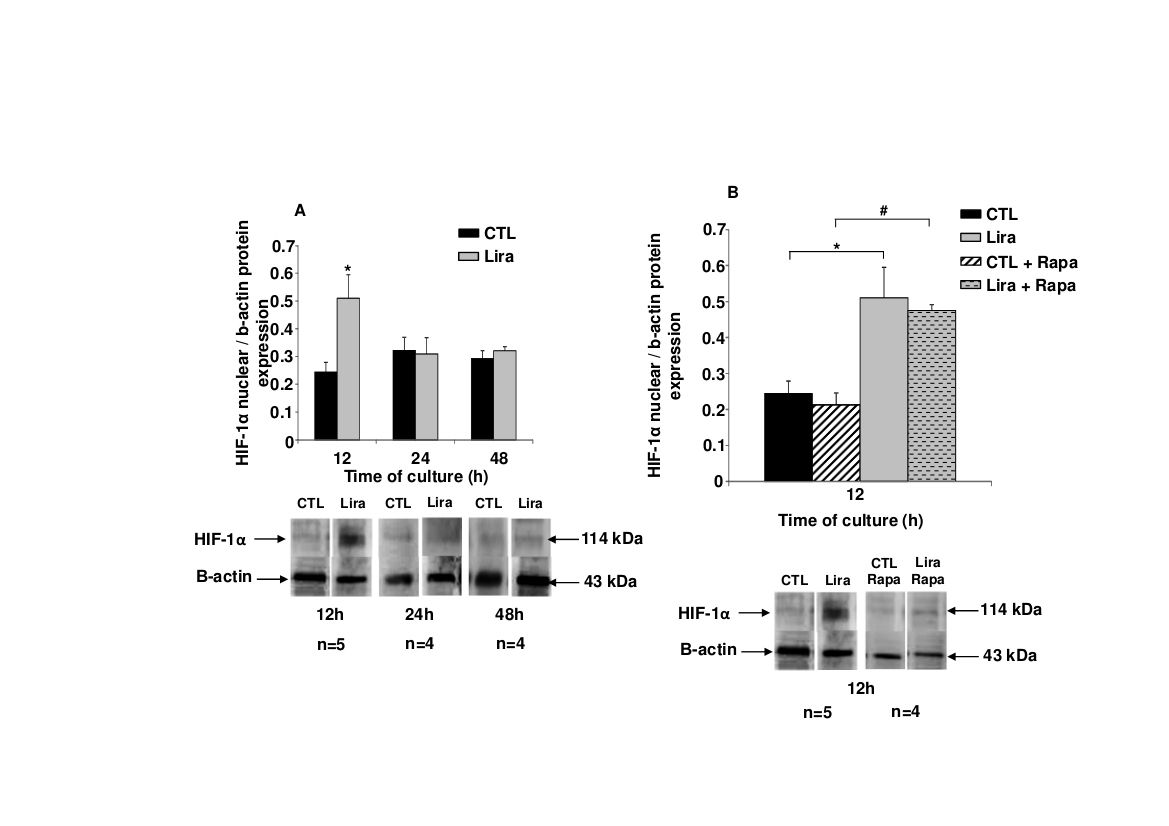

Supplement: S1 File — HIF-1α nuclear protein levels, determined by western blotting after 12, 24 and 48 h in culture. Black bars, control; grey bars, Lira (Figure A) HIF-1α nuclear protein levels, determined by western blotting after 12h in culture. Black bars, control; grey bars, Lira; striped bars, control + Rapa; grey bars with dotes, Lira + Rapa (Figure B). (TIF) [file pone.0147068.s001.tif]
